# Type 2 diabetes mellitus and risk of colorectal adenoma: a meta-analysis of observational studies

**DOI:** 10.1186/s12885-016-2685-3

**Published:** 2016-08-17

**Authors:** Feifei Yu, Yibin Guo, Hao Wang, Jian Feng, Zhichao Jin, Qi Chen, Yu Liu, Jia He

**Affiliations:** 1 Medical Service Research Division, Navy Medical Research Institute, Shanghai, China; 2 Department of Health Statistics, Second Military Medical University, No. 800 Xiangyin Road, Shanghai, 200433 China; 3 Department of Colorectal Surgery, Changhai Hospital, Shanghai, China; 4 College of Art & Science, University of San Francisco, San Francisco, USA

**Keywords:** Type 2 diabetes mellitus, Colorectal adenoma, Meta-analysis

## Abstract

**Background:**

To summarize the relationship between type 2 diabetes mellitus (T2DM) and risk of colorectal adenomas (CRA), we performed a meta-analysis of observational studies.

**Methods:**

To find studies, we searched PubMed, Embase, the Cochrane Library, Web of Science and conference abstracts and related publications for American Society of Clinical Oncology and the European Society of Medical Oncology. Studies that reported relative risks (RRs) or odds ratios (ORs) with 95 % confidence intervals (CIs) for the association between T2DM and risk of CRA were included. The meta-analysis assessed the relationships between T2DM and risk of CRA. Sensitivity analyses were performed in two ways: (1) by omitting each study iteratively and (2) by keeping high-quality studies only. Publication bias was detected by Egger’s and Begg’s tests and corrected using the trim and fill method.

**Results:**

This meta-analysis included 17 studies with 28,999 participants and 6798 CRA cases. We found that T2DM was a risk factor for CRA (RR: 1.52; 95 % CI: 1.29–1.80), and also for the advanced adenoma (RR: 1.41; 95 % CI: 1.06–1.87). Patients with existing T2DM (RR: 1.56; 95 % CI: 1.16–2.08) or newly diagnosed T2DM (RR: 1.51; 95 % CI: 1.16–1.97) have a risk of CRA. Similar significant results were found in retrospective studies (RR: 1.57; 95 % CI: 1.30–1.89) and population based cross-sectional studies (RR: 1.46; 95 % CI: 1.21–1.89), but not in prospective studies (RR: 1.27; 95 % CI: 0.77–2.10).

**Conclusions:**

Our results suggested that T2DM plays a risk role in the risk of developing CRA. Consequently, medical workers should increase the rate of CRA screening for T2DM patients so that they can benefit from behavioural interventions that can help prevent the development of colorectal cancer. Additional, large prospective cohort studies are needed to make a more convincing case for these associations.

**Electronic supplementary material:**

The online version of this article (doi:10.1186/s12885-016-2685-3) contains supplementary material, which is available to authorized users.

## Background

Diabetes mellitus (DM) is the fourth or fifth leading cause of death in developed countries and one of the biggest threats to human health worldwide [1]. More than 90 % of all DM is type 2 diabetes mellitus (T2DM) [2, 3]. Colorectal cancer (CRC) is the third most common cancer in the world. Colorectal adenoma (CRA) (also known as adenomatous polyp and always found by colonoscopy screen [4]) is a prevalent precancerous lesion that can lead to CRC through the adenoma–carcinoma sequence [5].

Research on risk factors for CRA has focused on several epidemiological factors, including smoking [6], alcohol consumption [5], body mass index [7], physical activity [8], and calcium intake [9]. Recent research on patients with diabetes suggested that insulin therapy and diabetes itself may increase the risk of CRC [10–12]. However, the association between T2DM and the risk of CRA risk has not yet been fully established. Some researchers asserted that there were no overall associations between T2DM and CRA risk [13–16], while others reported a higher risk [17–20]. To further examine these findings and provide evidence of association between T2DM and risk of CRA risk, we performed a meta-analysis about T2DM on the risk of CRA.

## Methods

### Literature search

Two investigators (FY and YG) independently conducted a systematic literature searches on January 10, 2016 in PubMed, Embase, the Cochrane Library and Web of Science without limiting the publication date range. The following search terms were used: (diabetes mellitus OR diabetes OR diabetic OR glucose) AND (colorectal OR colon OR rectal) AND (adenomas OR adenoma OR adenomatous OR polyp). No language restrictions and any other limitations were imposed. Conference abstracts on the websites of American Society of Clinical Oncology’s (ASCO) and the European Society for Medical Oncology’s (ESMO) annual meetings were also searched, along with the reference lists of the identified publications. Additional file 1 includes the complete searching process.

The titles and abstracts of all of the studies from the searches were screened independently by three reviewers (FY, YG and JF). To be included in this meta-analysis, studies had to be at least one of the following criteria: (1) retrospective or perspective observational study of the association between diabetes mellitus and CRA, or (2) a study reporting the relative risks (RRs) or odds ratios (ORs) for T2DM on CRA with 95 % confidence intervals (95 % CIs) adjusted for gender, age, or other factors. Studies reporting on the CRA recurrence were excluded.

### Data extraction

Data extraction was performed by three reviewers (FY, YG and WH), and verified independently for accuracy by a forth reviewer (JH). The following information was collected for each study: title and author, publication year, population, location, sample size, proportion of males and covariates controlled for by matching or multivariate analysis. For studies that reported several multivariate adjusted ORs, the effect estimate that adjusted for the maximum potential confounders was selected. Two investigators (FY and ZJ) conducted a quality assessment using the 9-star Newcastle-Ottawa Scale (NOS) [21], which was verified by a third investigator (YG). We considered studies with a NOS score of seven or more to be high-quality studies. The study selection process was based on the Meta-analysis of Observational Studies in Epidemiology (MOOSE) guidelines [22] and is described in Additional file 2.

### Statistical analysis

We examined the relationship between T2DM and CRA risk on the basis of the adjusted RRs and ORs and corresponding 95 % CI published in each study. A fixed effects model was used to estimate the pooled RR and OR with 95 % CIs if there was no evidence of heterogeneity; otherwise, a random effect model was used [23, 24]. Because the incidence of CRA is low, the ORs in retrospective studies approximate the RRs [25, 26]. Heterogeneity between the studies was evaluated by the chi-square test and I-squared (I^2^) statistic [23]. Statistical heterogeneity was considered significant when *p* < 0.10 [27].

Several methods were used to test and adjust for potential publication bias. Visual inspection of funnel plots was performed, and the Egger’s regression test [28] and Begg’s test [29] were used. Where publication bias existed, we used the trim and fill method to correct it [30]. Subgroups analyses by gender, adenoma subsite, and study type were performed to explore the potential heterogeneity among the included studies. Sensitivity analyses were performed in two ways: (1) by excluding each study iteratively from the meta-analysis and (2) by keeping high-quality studies only.

All statistical tests were two-sided and regarded as statistically significant at *p* < 0.05 Stata (Version 11.0; Stata Corp, College Station, TX) was used for all analyses.

## Results

### Study characteristics

**Fig. 1:**
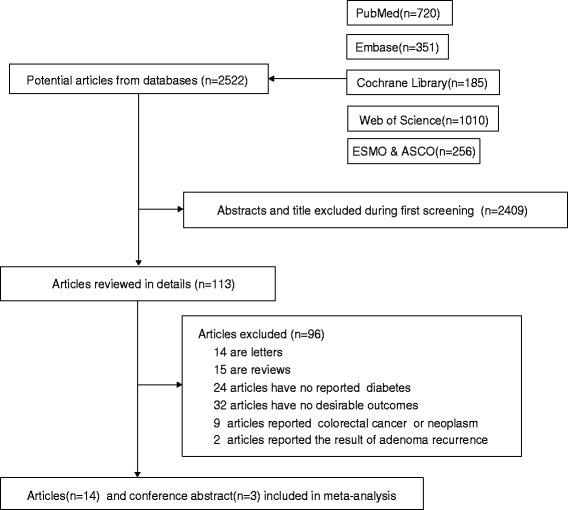
Flow chart of article selection process

Until January 10, 2016, 2522 records were retrieved by using our search strategy. After reviewing the titles and abstracts, 113 articles were further evaluated by reviewing the full texts. Of those remaining articles, we excluded studies that : (1) reported the data of adenoma recurrence were excluded [31, 32], (2) did not reported the RRs of getting CRA separately but mixed CRC and CRA patients [31], and (3) discussed the relationship between metformin [33] or insulin use [34] and CRA. We identified 17 studies that met all of our criteria [13–20, 35–44], including four conference abstracts [36, 37, 43, 44]. Figure 1 provides a flow chart of study selection. The final studies included 28,999 participants and 6798 CRA cases and 11 were rated as high-quality. Four of the conference abstracts rated less than seven stars due to insufficient information about their research. Table 1 includes the general characteristics of the included studies.

**Table 1:** Characteristic of studies included in the meta-analysis

Author	Year	Country	Study type	Mean age	Male (%)	Sample size	Category of exposure (*N*)	Outcome	Adjusted variable	NOS score
Chiranjeev Dash [13]	2014	US	retrospective	54.6 (8.5)	0 (0)	3668	T2DM (804)	CRA (917)	age, educational status, body mass index (weight (kg)/height (m)2), physical activity, family history of colorectal cancer in a first-degree relative, menopausal status, smoking status, alcohol intake, total energy intake, red meat intake, fruit and vegetable intake, and regular aspirin use	8
Heike Ursula [14]	2012	German	prospective	61.5	670 (62)	1554	T2DM (166)	Colorectal neoplasia (389)	age and sex	8
Tomomi Marugame [15]	2002	Japan	retrospective	52.4	1389 (100)	1389	Newly diagnosed T2DM (41)	CRA (560), Proximal adenomas(254), Distal adenomas (306)	hospital, rank in the Self Defense Forces, alcohol use, and cigarette smoking	7
Hongha T Vu [20]	2014	USA	retrospective	46	92 (36.8)	250	T2DM (125)	CRA (56)	ethnicity, body mass index, smoking, and alcohol use	7
Rodney Eddi [18]	2012	USA	retrospective	71	442 (56.4)	783	T2DM (89)	Adenomatous polyps (261)	Age, Sex, TG, LDL, HDL, Smoking, Family history of CRC, Aspirin, NSAID, Statins	7
Mehulkumar K. Kanadiya [19]	2013	American	retrospective	60.63(9.20)	1697 (49)	3465	T2DM (405)	CRA (852)	NA	3
Joseph Carl Anderson [35]	2011	USA	retrospective	NA	76 (38.0)	290	T2DM (46)	Any Sessile Serrated Adenomas (90)	NA	7
Bouwens, M [36]	2011	NA	retrospective	60	863	1836	T2DM	Combined adenoma-serrated phenotype (139)	NA	5^a^
de Kort, S [37]	2013	Netherlands	retrospective	NA	NA	3335	T2DM (326)	CRA (1112)	age, gender, BMI and other relevant risk factors	4^a^
Jill E. Elwing [38]	2006	US	retrospective	59.2	0 (0)	600	All diabetics (100)	Any Adenoma (159)	age, race, hypertension, hypercholesterolemia, BMI, and NSAID status	7
								Advanced adenoma (46)		
Kazushige Kawai [39]	2012	Japan	prospective	63.1(10.5)	109 (61.9)	176	T2DM (3888)	Polyp (69)	NA	7
Suminori Kono [40]	1998	Japan	retrospective	50–54	5193 (100)	5193	T2DM (166)	sigmoid colon adenomas (821)	body mass index (wt [kg]/ht [m]2), cigarette smoking, alcohol use, rank of the Self Defense Forces, and hospital.	7
Takasei Nishii [41]	2001	Japan	retrospective	48.4	951 (100)	951	T2DM (43)	Colon Adenomas(233)	Age- and BMI	6
Sunghwan Suh [42]	2011	Korea	retrospective	55.9	2528 (72.1)	3505	T2DM (509)	Multiple Adenomatous	sex, age, BMI, TC, HDL, TG, Fasting plasma glucose, HbA1c	7
								Polyps (509)		
Thomas R [43]	2012	NA	retrospective	58.4	1230 (95)	1295	T2DM (350)	Advanced adenoma (243)	NA	3^a^
Wang, JH [44]	2013	China	retrospective	NA	NA	470	T2DM	CRA(235)	abdominal circumference, daily calories & fat intake, increased diastolic blood pressure, history of hypertension or fatty liver, family history of cancer in digestive system, LDL and hsCRP, while female and daily fiber intake	6^a^
Misciagna, G [16]	2004	Italy	retrospective	57.5	154 (64.4)	239	Diabetes (34)/ Glucose (mg/100 ml)	CRA(153)	NA	8

*DM* diabetes mellitus, *T2DM* type 2 diabetes mellitus, *CRA* colorectal adenoma, *NSAID* nonsteroidal anti-inflammatory drugs, *TG* serum cholesterol and triglycerides, *BMI* body mass index, *HDL-C* high density lipoprotein cholesterol, *LDL-C* low density lipoprotein cholesterol, *hsCRP* high-sensitivity C-reactive protein, *T2DM* non-insulin dependentdiabetes mellitus, *TC* total cholesterol, *HDL* high-density lipoprotein, *NA* not available

^a^ conference abstract

### Diabetes and risk of colorectal adenoma

**Fig. 2:**
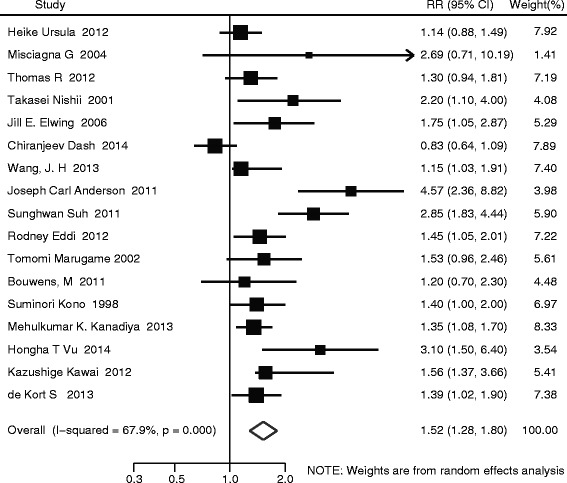
Forest plot of relative risk estimates of diabetes on risk of colorectal adenoma

The summary RR of diabetes on CRA was statistically significant (RR: 1.52; 95 % CI: 1.29–1.80). Evidence of the heterogeneity was identified (*I*^*2*^ = 65.6 %, *P* < 0.001). Figure 2 shows the results.

### Subgroup analysis

As shown in Table 2, we conducted subgroup analyses based on multiple factors, including sub-site of adenoma, geographic region, gender, and study type. The results showed that advanced adenoma was significantly associated with T2DM (RR: 1.41; 95 % CI: 1.06–1.87). However, a similar effect was not detected for proximal, distal, or colon adenoma. No evidence indicated significant associations between T2DM and CRA by gender, i.e., males (RR: 1.36; 95 % CI: 0.99–1.80) or females (RR: 1.29; 95 % CI: 0.76–2.17). he relationships between T2DM and CRA risk was significant in Europe (RR: 1.27, 95 % CI: 1.02–1.57), the USA (RR: 1.69; 95 % CI: 1.14–2.51) and Asia (RR: 1.57; 95 % CI: 1.21–2.05). A significant increase in risk was found in retrospective studies (RR: 1.57; 95 % CI: 1.30–1.89) and not in prospective studies (RR: 1.27; 95 % CI: 0.77–2.10).

**Table 2:** Subgroup analyses for the effect of diabetes on risk of colorectal adenoma

Subgroup	Sample size	RR (95 % CI)	*P* value	Heterogeneity		
				*χ* ^2^	I^2^	*P* value
Sub-site of adenoma						
Advanced adenoma	2145	1.41 (1.06–1.87)	0.018	1.50	0.0 %	0.473
Proximal adenoma	9343	1.28 (0.88–1.87)	0.199	10.89	72.4 %	0.012
Distal adenoma	9343	1.11 (0.89–1.38)	0.353	3.63	17.3 %	0.305
Colon adenoma	11201	1.06 (0.73–1.53)	0.758	10.72	72.0 %	0.013
Multiple adenoma	6840	1.95 (0.97–3.94)	0.061	6.73	85.2 %	0.009
Type of diabetes						
Known T2DM	20326	1.56 (1.16–2.08)	0.003	43.88	81.8 %	0.000
Newly diagnosed T2DM	1604	1.51 (1.16–1.97)	0.002	0.00	0.0 %	0.946
Gender						
Male	7839	1.33 (0.99–1.80)	0.059	4.74	36.7 %	0.192
Female	5135	1.29 (0.76–2.17)	0.348	10.33	80.6 %	0.006
Area						
Europe	13527	1.27 (1.02–1.57)	0.032	2.18	0.0 %	0.336
USA	5767	1.69 (1.14–2.51)	0.009	32.18	84.5 %	0.000
Asia	11684	1.57 (1.21–2.05)	0.001	13.23	62.2 %	0.021
Study type						
Prospective study	13871	1.27 (0.77–2.10)	0.357	11.93	83.2 %	0.003
Retrospective study	17405	1.57 (1.30–1.89)	0.000	25.40	60.6 %	0.005
Population based study	6122	1.46 (1.21–1.89)	0.005	2.06	3 %	0.357
Studies with high quality	26046	1.64 (1.26–2.14)	0.000	45.78	78.2 %	0.000

*T2DM* type 2 diabetes mellitus

### Sensitivity analysis

**Fig. 3:**
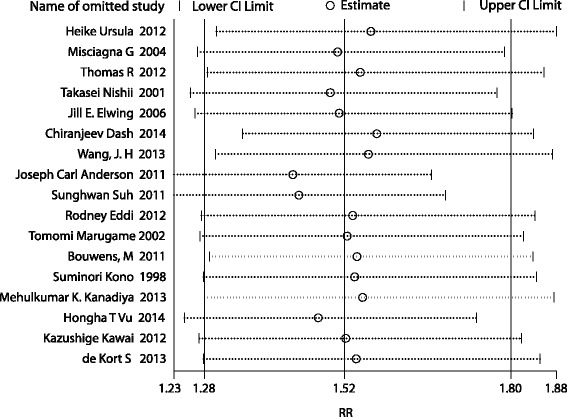
Result of sensitivity analyses by omitting one study in each turn

Sensitivity analysis indicated that no single study dramatically influenced the pooled RR. The results are shown in Fig. 3. Regardless of which study was omitted, the summary RRs were always greater than one. Similarly, Table 2 shows that excluding low-quality studies yielded results comparable with including all studies (RR: 1.64; 95 % CI: 1.26–2.14).

### Publication bias

**Fig. 4:**
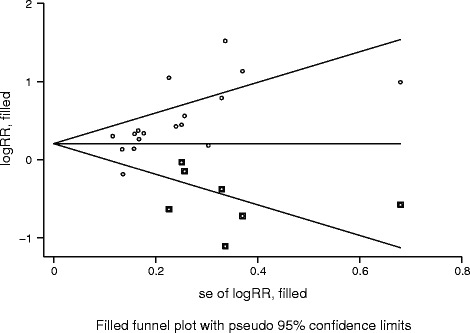
Filled funnel plot of log relative risk vs. standard error of log relative risks

The Begg’s rank correlation test (*p* = 0.001) and Egger’s regression test (*p* = 0.003) results showed potential publication bias that is described in Fig. 4. Once corrected by the trim and fill method [30], the result indicated that the pooled effect size did not changed.

## Discussion

This study indicated that patients with diabetes, especially type 2, have about 50 % increased relative risk of developing CRA than non-diabetic individuals, regardless of their geographic location. Although sample size was small in the newly diagnosed T2DM subgroup, the heterogeneity was also small and a significant risk relationship between T2DM and CRA was still detected. A similar result was only found in the advanced adenoma subgroup, not in the proximal, distal, colon or multiple adenoma subgroups. When low-quality studies were excluded, the positive association still existed. These results suggested that T2DM patients should pay more attention to their risk of CRA.

The positive relationship between T2DM and CRA may be linked to insulin resistance or an increased insulin-like growth factor 1 (IGF-1) might take effect in the adenoma–carcinoma process. High insulin levels could promote tumor growth [31, 45, 46]. Also, diabetes may lead to slower bowel transit, which would increase the probability of exposure to potential carcinogens for colonic mucosa [47–49]. It is worth noting that there might be some confounding effects because of the similar risk factors for both T2DM and CRA, such as physical inactivity, obesity, and an unhealthy diet habit [12, 50]. For example, a case–control study reported that higher red meat intake could significantly increase the risk of colon adenoma [51]. At the same time, obese people also tend to consume more red meats and have a higher risk of diabetes. Therefore, dietary habits might be a confounding factors. Finally, some researchers also report that obesity might be a confounder in the association between T2DM and colorectal disease [52].

Some studies reported a difference in risk between males and females [12, 39, 53–55]; however, the results of our subgroup analysis showed no difference. One possible explanation involves the redistribution of body fat that can occur when women experience menopause. The increase in visceral body mass fat could lead to hyperinsulinemia so that women, especially post-menopausal women, are more susceptible to colorectal diseases. However, the existence of menopause in some women cannot explain the different CRC risks for males and females [56–59]. Discrepancies among these studies and ours and the insignificant results by adenoma sub-site might be attributed to the limited sample sizes and insufficient statistical power. For the prospective studies, varied different follow-up procedures and mix of ethnicities different study populations might be the sources of heterogeneity.

Our analysis revealed that with T2DM have about a 5 % higher risk of CRA than newly diagnosed diabetes patients, revealing the duration of T2DM as a risk factor for CRA. A possible explanation is that known T2DM patients’ bowels are exposed to hyperinsulinemia or a hyperglycemic environment for a longer time, and such hormonal or metabolic abnormalities (according to former study [60]) could affect tumour growth. However, some studies reported that metformin use was a protective factor of CRA [33] and cancer [61]. If this is true, diagnosed diabetes patients should have a lower risk of adenomas than new patients, which is counter to our results. On the other hand, the severity of T2DM, which was not confirmed in the included studies, may affect colorectal disease risk and contribute to the mixed results. In sum, there might be a dose–response relationship between insulin and CRA, and further studies should include this as an important potential confounding factor.

Several limitations of in this meta-analysis that should be taken into consideration. First, results for several subgroups, such as gender and adenoma sub-site subgroup, were based on a limited number of studies. Therefore, we cannot rule out the possibility that insufficient statistical power is present. Second, in the present analysis, some small studies with inverse associations between T2DM and risk of CRA risk seemed to be suppressed. The presence of possible publication bias could have led to an overestimation of the effect of T2DM on CRA risk. However, the adjusted result was comparable after trim and fill method corrections. Third, we could not account for all of the confounding factors in the meta-analysis, though most confounders were adjusted in the original RRs. Many factors might induce the adenomas, such as age, ethnicity, inactivity, regular aspirin use, obesity, and family history of CRA, and menopausal status. We could not control for these covariates because of lack of relevant data. Relevant studies with additional data on these other factors may be found by searching by searching beyond the sources used for this study. Furthermore, we could not determine whether using insulin as a therapy for T2DM is an important factor because CRA risk might be altered by hyperinsulinemia, thought to be an important promoter of carcinogenesis [62, 63]. At the same time, metformin may have a direct anti-proliferative effect [64]. Finally, most of the existing studies did not discuss the influences of T2DM severity level on CRA risk. Thus, more cohort studies about these topics should be conducted.

## Conclusions

In conclusion, the results of our meta-analysis indicated that patients with T2DM have higher risks for the development of CRA, which is an important inducement for colorectal cancer. Our study has important implications for clinical and public health. Because T2DM and CRA are prevalent in the developed and developing countries [65], medical workers should increase the rate of CRA screening for T2DM patients so that they can benefit from behavioural interventions that can help prevent CRA [38]. Large prospective studies that investigate the interactions among environmental and behavioral factors, medications, and functional polymorphisms are also needed to further clarify the etiology of CRA.

## Additional files

Additional file 1: The detail searching process. (DOCX 14 kb) Additional file 2: The MOOSE list of this meta-analysis. (DOCX 19 kb)
