# Supplementary material for: Type 2 diabetes mellitus and risk of colorectal adenoma: a meta-analysis of observational studies
Source: BMC Cancer. 2016 Aug 17;16:642. doi: 10.1186/s12885-016-2685-3 (PMC4989384; doi:10.1186/s12885-016-2685-3)
Supplement: Additional file 1: — The detail searching process. (DOCX 14 kb) [file 12885_2016_2685_MOESM1_ESM.docx]

**Additional file 1: Searching process**

**Results of searching Pubmed:**

#1 rectum[All Fields] Items found: 65301

#2colon[All Fields] Items found: 175720

#3colorectal[All Fields] Items found: 122294

#4rectal[All Fields] Items found: 98035

#5 adenoma[MeSH Terms] Items found: 89263

#6 adenoma[All Fields] Items found: 89263

#7 adenomas[All Fields] Items found: 113721

#8 adenomatous [All Fields] Items found: 15817

#9 colorectal neoplasm[MeSH Terms] Items found: 159268

#10 colorectal neoplasms[MeSH Terms] Items found: 159268

#11 adenomatous polyposis of the colon[MeSH Terms] Items found: 5574

#12 rectal Neoplasms[MeSH Terms] Items found: 39985

#13 rectum neoplasm[MeSH Terms] Items found: 39985

#14rectum neoplasms[MeSH Terms] Items found: 39985

#15diabetes mellitus[MeSH Terms] Items found: 337566

#16 diabetes mellitus complications[MeSH Terms] Items found: 109357

#17 diabetes mellitus complication[MeSH Terms] Items found: 109357

#18 diabetes mellitus[All Fields] Items found: 386756

#19 #1 OR #2 OR #3 OR #4 Items found: 347275

#20 #5 OR #6 OR #7 OR #8 Items found: 119641

#21 #19 AND #20 Items found: 20515

#22 #9 OR #10 OR #11 OR #12 OR#13 OR #14 Items found: 159268

#23 #15 OR #16 OR #17 OR #18 Items found: 386756

#24 #21 OR#22 Items found:163363

#25 #23 AND #24 Items found:720

**Results of searching Cochrane:**

#1 "diabetes" (All text) Items found:38975

#2 "colorectal adenoma" (All text) Items found:427

#3 #1 AND #2 185

**Results of searching Embase:**

#1 'rectum'/exp OR 'rectum' Items found: 139,215

#2 'colon'/exp OR 'colon' Items found: 319161

#3 'colorectal' Items found: 194,534

#4rectal Items found: 117768

#5 Rectum Items found:139215

#6 adenomas Items found: 37956

#7 adenomatous Items found: 19277

#8 adenoma Items found: 93365

#9 neoplasm Items found:652375

#10 neoplasms Items found: 197057

#11polyposis Items found: 21273

#12diabetes mellitus Items found: 682339

#13 diabetes Items found:823179

#14 #1 OR #2 OR #3 OR #4 OR #5 Items found: 139215

#15 #6 OR #7 OR #8 OR #9 OR #10 OR #11 Items found:885714

#16 #12 OR #13 Items found: 823179

#17 #14 AND #15 AND #16 Items found: 351

**Results of searching Web of Science:**

TS=(adenoma* OR　neoplasm* OR polyp*) AND TS=(diabetes mellitus) AND TS=(colorectal OR colon OR rectum OR rectal)

Items found:1010

**Results of searching ESMO:**

Using “colorectal adenoma diabetes”, Items found: 251

**Results of searching ASCO:**

Using "adenoma" AND "colorectal" AND "diabetes" in abstract, Items found: 5
