# Supplementary material for: Type 2 diabetes mellitus and risk of colorectal adenoma: a meta-analysis of observational studies
Source: BMC Cancer. 2016 Aug 17;16:642. doi: 10.1186/s12885-016-2685-3 (PMC4989384; doi:10.1186/s12885-016-2685-3)
Supplement: Additional file 2: — The MOOSE list of this meta-analysis. (DOCX 19 kb) [file 12885_2016_2685_MOESM2_ESM.docx]

Type 2 diabetes mellitus and risk of colorectal adenoma: a meta-analysis of observational studies

Feifei Yu^1§^, Yibin Guo^1§^, Hao Wang^2§^, Jian Feng^1§^, Zhichao Jin ^1^, Qi Chen^1^, Yu Liu^3^, Jia He^1*^

**Author Affiliations:**

^1^Department of Health Statistics, Second Military Medical University, Shanghai, China

^2^Department of Colorectal Surgery, Changhai Hospital, Shanghai, China

^3^College of Art & Science, University of San Francisco

^§^These authors contributed equally and are co-first authors of this article.

***Corresponding author:** Prof. Jia He, Department of Health Statistics, Second Military Medical University, No. 800 Xiangyin Road, Shanghai 200433, China

Tel: +86-21-81871441, Fax: +86-21-81871441, E-mail: [hejia63@yeah.net](mailto:hejia63@yeah.net)

**Key words:** type 2 diabetes mellitus, colorectal adenoma, meta-analysis

**MOOSE Statement - Reporting Checklist for Authors, Editors, and Reviewers of Meta-analyses of Observational Studies**

| **Reporting Criteria** | **Reported (Yes/No)** | **Reported on Page** |
| --- | --- | --- |
| **Reporting of background should include** | | |
| Problem definition | YES | 3 |
| Hypothesis statement | YES | 3 |
| Description of study outcomes | YES | 3-4 |
| Type of exposure or intervention used | YES | 4 |
| Type of study designs used | YES | 4 |
| Study population | YES | 4 & Table 1 |
| **Reporting of search strategy should include** | | |
| Qualifications of searchers (eg librarians and investigators) | YES | 3 |
| Search strategy, including time period used in the synthesis and key words | YES | 4 |
| Effort to include all available studies, including contact with authors | YES | 4 |
| Databases and registries searched | YES | 4 |
| Search software used, name and version, including special features used (eg explosion) | NO |  |
| Use of hand searching (eg reference lists of obtained articles) | YES | 4 |
| List of citations located and those excluded, including justification | YES | 4-5 & Figure1 |
| Method of addressing articles published in languages other than English | YES | 4 |
| Method of handling abstracts and unpublished studies | YES | 4 |
| Description of any contact with authors | NO |  |
| **Reporting of methods should include** | | |
| Description of relevance or appropriateness of studies assembled for assessing the hypothesis to be tested | YES | 4-5 |
| Rationale for the selection and coding of data (eg sound clinical principles or convenience) | YES | 4-5 |
| Documentation of how data were classified and coded (eg multiple raters, blinding and interrater reliability) | YES | 5 |
| Assessment of confounding (eg comparability of cases and controls in studies where appropriate) | YES | 5 |
| Assessment of study quality, including blinding of quality assessors, stratification or regression on possible predictors of study results | YES | 5 |
| Assessment of heterogeneity | YES | 4-5 |
| Description of statistical methods (eg complete description of fixed or random effects models, justification of whether the chosen models account for predictors of study results, dose-response models, or cumulative meta-analysis) in sufficient detail to be replicated | YES | 3-5 |
| Provision of appropriate tables and graphics | YES | 5 |
| **Reporting of results should include** | | |
| Graphic summarizing individual study estimates and overall estimate | YES | Figure 2-4 |
| Table giving descriptive information for each study included | YES | Table 1 |
| Results of sensitivity testing (eg subgroup analysis) | YES | 6,  Table 2 |
| Indication of statistical uncertainty of findings | YES | 6 |
| **Reporting of discussion should include** | | |
| Quantitative assessment of bias (eg publication bias) | YES | 6-7, Figure4 |
| Justification for exclusion (eg exclusion of non-English language citations) | YES | 6-8 |
| Assessment of quality of included studies | YES | 6-8 |
| Strengths and weaknesses | YES | 7-8 |
| **Reporting of conclusions should include** | | |
| Consideration of alternative explanations for observed results | YES | 9 |
| Generalization of the conclusions (eg appropriate for the data presented and within the domain of the literature review) | YES | 9 |
| Guidelines for future research | YES | 9 |
| Disclosure of funding source | YES | 10 |

NA: Not Applicable
